# Automated Monitoring System for Suspended Photocatalytic Batch Reactions Based on Online Circulatory Spectrophotometry

**DOI:** 10.3390/nano14060508

**Published:** 2024-03-12

**Authors:** Da-Peng Lei, Jian-Hua Huang

**Affiliations:** 1 Department of Chemistry, Zhejiang Sci-Tech University, Hangzhou 310018, China; photocatalyst@163.com; 2 Wenzhou Quality and Technology Testing Research Institute, Wenzhou 325007, China

**Keywords:** automated monitoring system, batch suspended photocatalytic reaction, online circulatory spectrophotometry, kinetic study

## Abstract

Employing an automated monitoring system (AMS) for data acquisition offers benefits, such as reducing the workload, in the kinetic study of suspended photocatalytic batch reactions. However, the current methods in this field tend to narrowly focus on the substrate and often overlook the optical characteristics of both the mixture and solid particles. To address this limitation, in this study, we propose a novel AMS based on online circulatory spectrophotometry (OCS) and incorporate debubbling, aeration, and segmented flow (DAS), named DAS-OCS-AMS. Initially, a debubbler is introduced to mitigate the issue of signal noise caused by bubbles (SNB). Subsequently, an aerated and segmented device is developed to address the issue of particle deposition on the inner wall of the pipeline (PDP) and on the windows of the flow cell (PDW). The proposed DAS-OCS-AMS is applied to monitor the kinetics of the photocatalytic degradation of Acid Orange Ⅱ by TiO_2_ (P25), and its results are compared with those obtained using the traditional OCS-AMS. The comparative analysis indicates that the proposed DAS-OCS-AMS effectively mitigates the influence of SNB, PDP, and PDW, yielding precise results both for the mixture and solid particles. The DAS-OCS-AMS provides a highly flexible universal framework for online circulatory automated monitoring and a robust hardware foundation for subsequent data processing research.

## 1. Introduction

The kinetic study of suspended photocatalytic batch reactions (SPBRs) is of great significance for developing new and improved photocatalytic materials and chemical kinetic models [1], as well as for optimizing the design and operation of photocatalytic reactors [2,3]. One of the primary objectives of photocatalytic kinetic studies is the evaluation of photocatalytic activity. This is because not only are the related research activities centered around obtaining higher-performance new materials but photocatalytic activity is also one of the four critical features that need to be reported [1]. SPBR refers to a photocatalytic reaction conducted in a suspended state in a batch, allowing for an increased contact area between the photocatalyst and the substrate, thereby enhancing the efficiency of reactions [4,5]. However, the heterogeneity [6], polydispersity [7], and multi-component characteristics [8,9] of SPBRs pose challenges in acquiring kinetic data. Therefore, the data acquisition methods for the kinetic study of SPBRs have gained considerable attention, and accordingly, various automated monitoring systems (AMSs) have been developed to reduce the workload and enhance the efficiency and accuracy.

In recent years, research on AMSs for the kinetic data acquisition of SPBRs based on online automated sample pretreatment techniques has demonstrated strong performance compared to traditional manual methods. For instance, Salazar-Beltrán et al. [10] combined sequential injection analysis (SIA) with filtration and high-performance liquid chromatography (HPLC) to monitor the TiO_2_ photocatalytic degradation of phthalates. Similarly, Guevara-Almaraz et al. [11] proposed a multisyringe chromatography system with an online filtration device to automate the monitoring of the photocatalytic degradation of the mixtures of isoniazid and pyrazinamide using TiO_2_ or ZnO, respectively. In contrast, Koichiro Masuda and Shū Kobayashi [6] utilized robotically manipulated probes for automated sampling and ionization spray mass spectrometry to monitor hydroxymethylation reactions. These approaches separate the reaction and detection, facilitate adjustments to the experimental conditions, and can integrate different detection technologies to obtain information on substrates and their intermediates. However, the requirement for sampling and pretreatment, as well as the inability to obtain kinetic information for solid particles, represents a drawback of these methods. The drawbacks of sampling and the absence of information on solid particles may affect the understanding of the reaction mechanisms. This is due to the fact that the optical properties of suspended solid particles, based on both particle density and aggregation state, are significant for the understanding of reaction mechanisms [7,12,13].

To address the limitations of sampling and pretreatment, recent research has focused on in situ real-time monitoring, involving direct spectral monitoring of the reactor using various optical methods to acquire kinetic data without sampling. For instance, Tapia-Tlatelpa et al. [14] monitored the photocatalytic degradation kinetics of dyes on TiO_2_ using light scattering spectroscopy. In contrast, Rößler et al. [15] achieved the automated monitoring of photo-induced synthesis reactions through in situ Raman and UV-Vis spectroscopy probes. Additionally, Keller et al. [16] used a smartphone combined with a Python-written data processing program to obtain kinetic data on dye adsorption and photocatalytic degradation through changes in the digital image’s RGB intensity. Similarly, Danyliuk et al. [17,18] used a smartphone to measure the photodegradation rate. Furthermore, Fu et al. [19] used a diamond prism to achieve total reflection infrared tracking of the liquid–solid interface in photochemical reactions. These in situ monitoring methods provide valuable references for the development of new technologies, offering the advantage of no need for sampling. However, they also have limitations, including the potential for signal interference from factors such as stirring and excitation light [20]. Although these in situ monitoring methods had the opportunity to pay attention to the optical properties of solid particles, this was overlooked.

Notably, there has been widespread attention on improvements in in situ monitoring using spectrophotometry due to its high applicability and sensitivity in routine laboratory analyses [8,9]. For example, Ditrói et al. [21] developed an external device that integrates the reaction and detection using fiber-optic-connected light sources and spectrophotometers. In contrast, Bukman et al. [22] converted a cuvette into a microreactor, and Tatarchuk et al. [23] similarly adopted this approach. These methods eliminate the need for a sampling step and offer the advantages of high time resolution and minimal sample usage. In addition to the previously mentioned issues with in situ monitoring methods, there may also be challenges related to reduced flexibility in experimental operation and the deposition of solid particles on the reactor wall [1,12]. However, it is important to note that while the aforementioned works have significantly contributed to acquiring kinetic data from SPBRs, they primarily focus on the substrate. None of them simultaneously take into account the optical properties of solid particles, broad experimental conditions, and operational simplicity, despite the great significance of these aspects for kinetic studies. Therefore, an essential challenge remains in simultaneously considering these three aspects.

In this study, to simultaneously satisfy these three aspects, we propose a novel AMS, named DAS-OCS-AMS, which is based on online circulatory spectrophotometry (OCS) and incorporates debubbling, aeration, and segmented flow (DAS). The OCS, as a simple online circulation method, has been widely used to monitor the absorbance in homogeneous Fenton reactions [24,25]. Initially, OCS-AMS was used to directly monitor the absorbance of the mixed suspension. This method acquires absorbance at different wavelengths and separates the reaction and detection, providing the benefits of easy experimental operation and minimal interference with monitoring signals caused by the excitation light and stirring. However, this approach encountered problems, including signal noise from bubbles (SNB) [26], and solid particle deposition on the inner wall of pipelines (PDP) and on the windows of a flow cell (PDW) [14,27,28]. SNB refers to the sudden increase in signal intensity of absorbance caused by the presence of bubbles during monitoring. PDP and PDW refer to the spontaneous adhesion of particles to the inner surfaces of the pipelines [27,28] and the windows of the flow cell, respectively. To address the SNB problem, a debubbler was developed to achieve separation through gravity by utilizing the density disparity between the gas and the liquid. To address the issues of the PDP and PDW, we propose an aerated and segmented device (ASD). The device is designed to generate high-velocity slug flow to eliminate the PDP and PDW, as well as to incorporate segmented flow to decrease the total circulation time for enhancing the effect in preventing PDP and PDW [29,30]. The segmented flow analysis (SFA) was first proposed by Skeggs in 1957 [31]. Its basic principle involves the use of discrete segments separated by gas for precise mixing, reaction, and monitoring [32,33].

In summary, the development and validation of the DAS-OCS-AMS involves both hardware and data processing aspects. This paper primarily focuses on the hardware aspect. In comparison to the traditional OCS-AMS, the significant contributions of this study can be summarized as follows:

1. The development of the debubbler to address the SNB problem;
2. The development of the ASD to minimize the influence of the PDP and PDW;
3. The construction of the DAS-OCS-AMS to provide a generic framework of online automated monitoring using various spectroscopy techniques for the SPBRs.

## 2. Materials and Methods

### 2.1. Materials

The activated carbon (AC) was sourced from Sinopharm Chemical Reagent Co., Ltd. (Shanghai, China). Graphitic carbon nitride (g-C_3_N_4_) was prepared through thermal polymerization of urea, as previously reported [34]. Briefly, the synthesis was performed by heating 10 g of urea in a covered crucible inside a muffle furnace at 550 °C for 180 min, with a heating rate of 10 °C/min. Subsequently, the furnace was allowed to cool down to room temperature. Both the AC and g-C_3_N_4_ were sieved through a 100-mesh sieve prior to use. TiO_2_ (P25) was from Degussa Chemicals (Hanau, Germany). Acid Orange II (AOII) was from Shanghai Makclin Biochemical Co., Ltd. (Shanghai, China). The water used in this study was obtained from Wenzhou Angel Space Water Co., Ltd. (Wenzhou, China) and had a resistivity greater than 0.25 MΩ·cm and a pH value of 5.9 ± 0.1. Aqueous stock solution of AOII was prepared at mass concentration of 2.00 g/L.

### 2.2. Method

**Figure 1:**
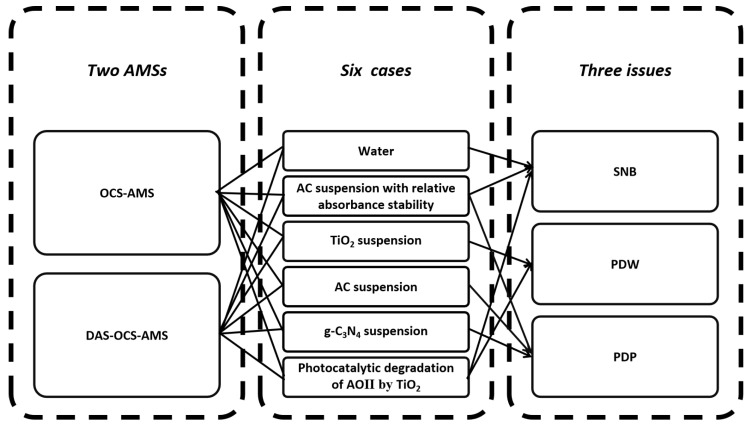
The schematic of this work.

A schematic of the study is outlined in Figure 1. As shown in the figure, this study compares the DAS-OCS-AMS with the traditional OCS-AMS across six cases. The objective is to evaluate their performance in terms of SNB, PDW, and PDP elimination.

#### 2.2.1. The Proposed DAS-OCS-AMS

**Figure 2:**
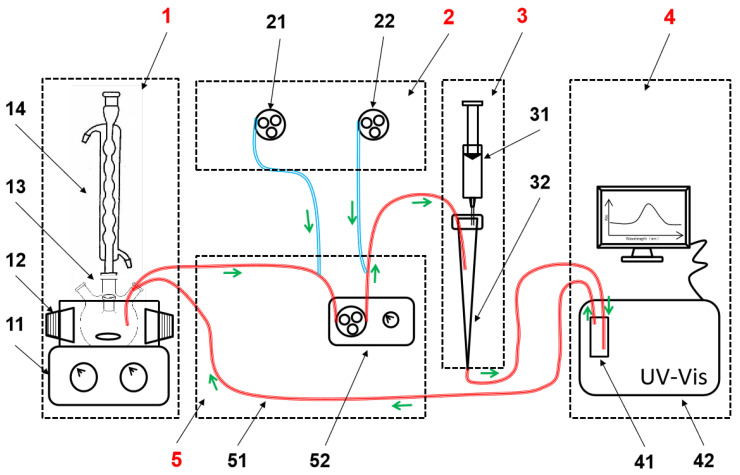
The schematic diagram of the DAS-OCS-AMS, includes the following components: 1—Reaction unit, 11—stirrer, 12—LED lamps, 13—reactor, 14—condenser tube, 2—aerated and segmented unit, 21—drainage peristaltic pump, 22—aerated peristaltic pump, 3—debubble unit, 31—pipette tip, 32—syringe, 4—detection unit, 41—flow cell, 42—UV-Vis spectrophotometer, 5—online circulation unit, 51—PTFE tubing, 52—circulation peristaltic pump.

A schematic diagram of the DAS-OCS-AMS is depicted in Figure 2. As shown in the figure, the DAS-OCS-AMS includes five core units. The reaction unit provides the necessary conditions and environment for the chemical reaction. The aerated and segmented unit creates a stable detection period for the detector and clears the solid particles, bubbles, and liquid from the pipeline after each detection. This unit mainly consists of two constant-speed peristaltic pumps (CKP-DC-S08, Kamoer Fluid Tech (Shanghai) Co., Ltd., Shanghai, China, with flow rates of 81 mL/min for aeration and 64 mL/min for drainage), an intermittent timer (B14M, Quanzhou Innovator Electric Appliance Group Co., Ltd., Quanzhou, China), and two time-delay relays (H3Y-2, Shenxintai Corporation, Shenzhen, China). The debubble unit is used to separate bubbles from the fluid to ensure that the bubbles do not interfere with the monitoring signal, mainly including a 5 mL pipette tip and a 60 mL syringe with a fixed base. The detection unit is responsible for monitoring the absorbance, including a UV-Vis spectrophotometer (TU-1810PC, Beijing Purkinje General Instrument Co., Ltd., Beijing, China) and a flow cell (1 cm path length, 0.48 mL, JGS1, Jiangsu Yixing Hongjun Optical instrument equipment Factory, Yixing, China). The online circulation unit ensures the circulation of the reaction solution and mainly consists of 2.0 mm ID PTFE tubing and an adjustable-speed peristaltic pump (NEW KCP, Kamoer Fluid Tech (Shanghai) Co., Ltd., Shanghai, China). The red and blue lines represent the paths of the liquid and gas in the system, respectively, while the green arrows indicate the direction of fluid flow.

#### 2.2.2. The Proposed Debubbler

**Figure 3:**
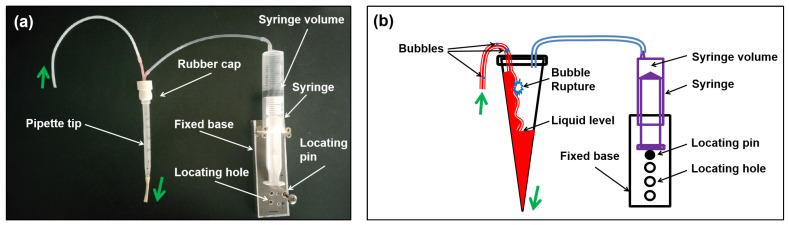
(**a**) The physical representation and (**b**) operational principle of the debubbler.

Bubbles have always presented a challenge for optical detection in flow analysis systems [28]. Therefore, it is essential to take specific measures to address the bubble issue. For this purpose, several methods are available to mitigate the bubble problem, including installing a debubbler, using an electronic bubble valve, employing a custom-made bubble-free flow cell, and adjusting experimental conditions [28]. One of the objectives of this study is to ensure the widest possible range of experimental conditions. Therefore, we proposed a debubbler inspired by the vertical gravity gas–liquid separator for separating bubbles from the fluid [29]. The detailed structure and principles of the proposed debubbler are illustrated in Figure 3a and b, respectively.

As depicted in Figure 3a, the debubbler comprises a pipette tip, rubber cap, syringe, and its fixed base. Figure 3b demonstrates the downward flow of the liquid along the inner wall of the pipette tip due to gravity as it traverses the debubbler, leading to the rupture of bubbles caused by the significant density differential between the liquid and gas phases. As the liquid accumulates at the bottom and its level rises, the internal pressure of the debubbler gradually increases until it reaches equilibrium. Experimental conditions, such as circulation velocity and temperature, may impact the liquid level. Therefore, this study regulated the liquid level by adjusting the internal space of the debubbler using a syringe.

#### 2.2.3. The Proposed ASD

In the automated monitoring of online circulatory flow for SPBRs, the presence of the PDP and PDW poses a significant challenge [28]. Therefore, it is necessary to address this issue with minimal disturbance to the reaction process. Inspired by our daily observations of pipeline cleaning, we discovered that abrupt changes in flow velocity or the introduction of slug flow can clean the PDP. Additionally, we gained insights from relevant studies in fluid mechanics on the impact mechanism of slug flow on pipeline cleaning [35]. Moreover, also inspired by the SFA [29,30], the utilization of segmented flow can reduce the total online circulation time, thereby enhancing its effectiveness in preventing PDP and PDW. Therefore, we proposed an innovative segmented flow and aeration cleaning strategy and developed a corresponding ASD.

**Figure 4:**
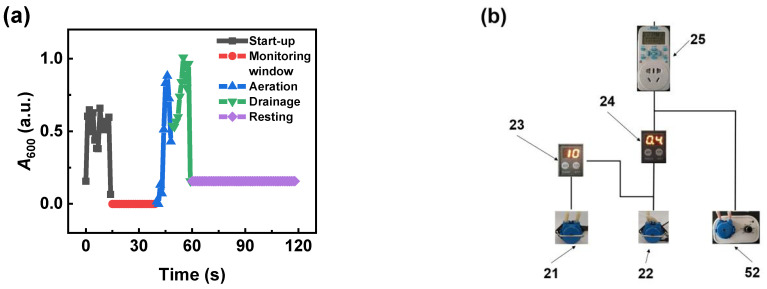
(**a**) The time-dependent absorbance signal sample of water within one working cycle of the ASD. (**b**) The circuit connection of the ASD includes the following additional components: 21—drainage peristaltic pump, 22—aerated peristaltic pump, 23—drainage time-delay relay, 24—aeration time-delay relay, 25—intermittent timer, 52—circulation peristaltic pump.

Figure 4a depicts a time-dependent absorbance signal sample of water during a working cycle of the ASD. The experimental parameters include the time scanning mode of the spectrophotometer (recording once per second), the on/off time of the intermittent timer (59 s and 61 s), and the delay time for the aeration and drainage peristaltic pumps (40 s and 10 s). Based on these parameters, we can deduce that the time resolution (*TR*) is 2 min, the aeration duration (*AD*) is 10 s, the drainage duration is 9 s, and the resting duration is 61 s.

Figure 4b details the circuit connections between the peristaltic pumps and timers of the ASD. This control circuit is designed to automate the segmentation process and create high-speed slug flow. As depicted in the figure, the working process of the ASD involves five distinct stages: start-up, monitoring window, aeration, drainage, and resting. The working principle is as follows: Firstly, the intermittent timer initiates the online circulation task of the peristaltic pump at preset time intervals. During the monitoring window stage, the detector promptly conducts monitoring. After the monitoring is completed, the aeration peristaltic pump is activated by the intermittent timer to pump air into the circulation pipeline, creating a high-speed slug flow that promptly cleans the deposited solid particles and bubbles. Subsequently, the drainage peristaltic pump is activated to pump air to replace the liquid for circulation, completing the discharge of the liquid. Finally, the intermittent timer shuts down all the peristaltic pumps, putting the device into a resting state, ready for the next working cycle to begin.

#### 2.2.4. The Traditional OCS-AMS

As outlined in Figure 2, the traditional OCS-AMS consists of a reaction unit, a detection unit, and an online circulation unit [24,25].

### 2.3. Case Studies

As shown in Figure 1, this study utilized six case studies to achieve different research objectives. Firstly, water was selected to assess its performance in preventing SNB, as bubbles are primarily enveloped by and closely interact with water. For detailed experimental conditions and parameter settings for the water case studies, please refer to Table S1 in the Supplementary Materials.

Secondly, the AC aqueous suspension with relatively stable absorbance, which was stirred for a long time (over 24 h), was chosen as the benchmark to evaluate the performance in preventing PDP. This is because there is no significant PDW for AC suspension, and after a long time stirring, the particle deposition on the inner wall of the reactor reaches equilibrium, providing an effective basis for evaluating the performance in preventing PDP.

Thirdly, we employed three aqueous suspensions of AC, g-C_3_N_4_, and TiO_2_ to assess the performance of the ASD in preventing PDP and PDW. These suspensions were selected for their different properties and particle sizes, which could lead to varied experimental results. It is worth noting that we selected the TiO_2_ suspension because, for the DAS-OCS-AMS, this suspension represents the most challenging one. Specifically, at a pH of 6, it is close to the isoelectric point of 6.2 and has an average hydrodynamic diameter between 1 and 10 µm [36,37]. This suspension shows strong PDW, providing valuable visual opportunities to evaluate the performance in preventing PDW. For detailed experimental conditions and parameter settings for the case studies involving pure solid aqueous suspensions, please refer to Table S2 in the Supplementary Materials.

Additionally, we chose the photocatalytic degradation of AOII by TiO_2_ as a case study because the addition of anionic dye AOII has almost no effect on the pH of TiO_2_ suspension, and at this pH, TiO_2_ does not strongly adsorb AOII, thus maintaining a strong interaction between TiO_2_ and AMS. For detailed experimental conditions and parameter settings, please refer to Table S3 in the Supplementary Materials.

## 3. Results and Discussion

### 3.1. Results for the Water

In this section, water is employed as a case study. Firstly, a comparison of the signal stability was conducted between the OCS-AMS and the DAS-OCS-AMS under severe reaction conditions. Subsequently, the mechanism of SNB generation in the OCS-AMS was analyzed. Finally, in order to enhance the signal stability of DAS-OCS-AMS, the effects of four main parameters were analyzed.

#### 3.1.1. Comparison of Signal Stability under Two Severe Experimental Conditions

**Figure 5:**
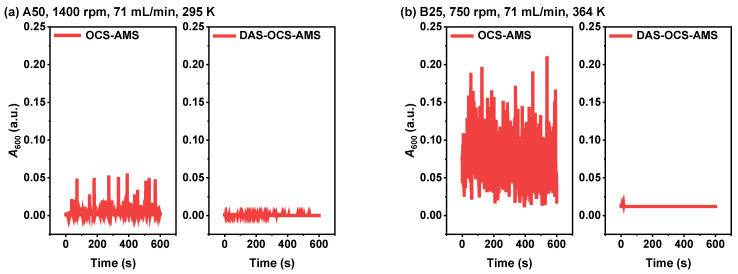
Time-dependent *A*_600_ of water under two severe experimental conditions by the OCS-AMS and the DAS-OCS-AMS: (**a**) utilizing stir bar A50 at the maximum stirring speed of 1400 rpm, circulation velocity of 71 mL/min, and a temperature of 295 K; (**b**) utilizing stir bar B25 at a stirring speed of 750 rpm, circulation velocity of 71 mL/min, and a temperature of 364 K.

To assess the performance of the proposed DAS-OCS-AMS in preventing SNB compared with the OCS-AMS, Figure 5 depicts the time-dependent absorbance at 600 nm (*A*_600_) under two severe experimental conditions obtained using these two AMSs. As illustrated in the figure, the results obtained by the OCS-AMS show very high-density SNB under both conditions. In contrast, the results obtained by the DAS-OCS-AMS show no significant SNB. This is attributed to the debubbler of the DAS-OCS-AMS, which has the capability to achieve gas–liquid separation by leveraging the density difference between the gas and liquid. These results indicate that the proposed debubbler exhibits exceptional performance in eliminating SNB. The adoption of this technology has the potential to significantly enhance the robustness of online circulatory monitoring for the homogenous system.

#### 3.1.2. Analysis of the Mechanism of SNB Generation in the OCS-AMS

**Figure 6:**
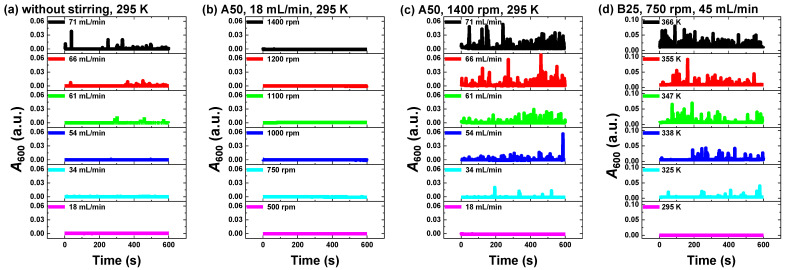
The time-dependent *A*_600_ of water under different experimental conditions by the OCS-AMS: (**a**) increasing circulation velocity at 295 K without stirring; (**b**) increasing stirring speed at the lowest circulation velocity (18 mL/min) at 295 K; (**c**) increasing circulation velocity at the maximum stirring speed (1400 rpm) at 295 K; and (**d**) increasing temperature at a fixed stirring speed (750 rpm) and circulation velocity (45 mL/min).

To gain insights into the mechanism of SNB generation in the OCS-AMS, Figure 6 presents the results of the influence of circulation velocity, stirring speed, and temperature on the time-dependent *A*_600_ of water by the OCS-AMS. In Figure 6a, it is evident that the onset of SNB begins when the circulation velocity reaches a certain threshold, and a higher circulation velocity leads to an earlier onset of SNB. For instance, at a circulation velocity of 61 mL/min, SNB begins to appear at 295 s, while at 71 mL/min, the appearance of SNB is notably earlier than at 61 mL/min. This can be attributed to the fact that a faster peristaltic pump speed generates greater negative pressure [38], resulting in an earlier occurrence of SNB. Figure 6b reveals that at the lowest circulation velocity (18 mL/min), SNB does not appear, even with the highest stirring speed (1400 rpm).

Comparative analysis of Figure 6a–c indicates a synergistic effect of circulation and stirring on SNB generation. For instance, at a maximum stirring speed of 1400 rpm and a circulation velocity of 34 mL/min, the appearance of SNB is observed. This is likely due to the increase in SNB density as the circulation velocity rises, with small bubbles being drawn into the circulation pipeline more readily and accumulating more rapidly. Figure 6d demonstrates that at a specific circulation velocity and stirring speed, the density of SNB increases with temperature. This is possibly due to the higher temperature, leading to an increase in the density of small bubbles in the reactor, resulting in a higher density of SNB.

In summary, circulation velocity, stirring speed, and temperature are pivotal factors influencing the generation of SNB, and they have a synergistic effect. This is due to the positive correlation between the density of bubbles in the reactor with stirring intensity and temperature. Additionally, the accumulation rate of bubbles in the pipeline is positively correlated with the density of bubbles in the reactor and the circulation velocity, suggesting that increasing experimental conditions may lead to an increase in SNB density. Therefore, the addition of an SNB elimination device is necessary to ensure the robustness of the monitoring signal and facilitate the adjustment of experimental conditions.

#### 3.1.3. Influence of Parameters on the Operational Stability of the Debubbler

**Figure 7:**
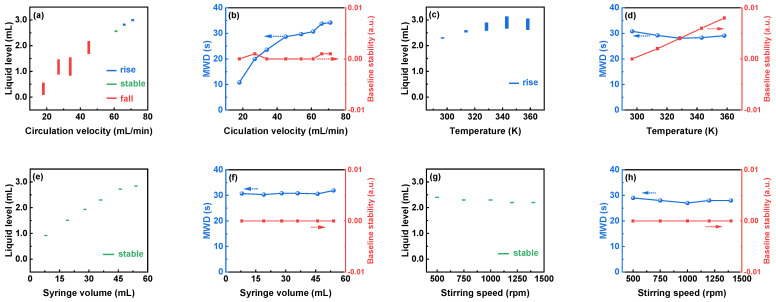
The liquid level and its stability, the MWD, and the baseline stability when the levels of (**a**,**b**) circulation velocity, (**c**,**d**) temperature, (**e**,**f**) syringe volume, and (**g**,**h**) stirring speed are increased, respectively.

To enhance the signal stability of DAS-OCS-AMS, Figure 7 depicts the effects of four parameters (circulation velocity, temperature, syringe volume, and stirring speed) on the operational stability of the debubbler, encompassing liquid level, liquid-level stability, MWD, and baseline stability. As depicted in the figure, circulation velocity, temperature, and syringe volume exert a noteworthy influence on the liquid level, while the impact of stirring speed is comparatively minimal. Furthermore, the MWD is predominantly affected by the circulation velocity, whereas baseline stability is solely influenced by temperature.

Figure 7a,b indicate that an escalation in circulation velocity results in an elevation in the liquid level, showcasing optimal stability. This phenomenon could be linked to the equilibrium between circulation velocity and the internal pressure of the debubbler. Furthermore, Figure 7c,d illustrate that with increasing temperature, the liquid level, its stability, and the baseline stability exhibit an upward trend. This could be attributed to the alterations in fluid properties induced by temperature, causing a rise in the density of small bubbles. This underscores the necessity of considering the influence of temperature during online monitoring and emphasizes the importance of calibrating the baseline at the same temperature to ensure the accuracy and stability of monitoring results. Additionally, Figure 7e,f demonstrate that augmenting the syringe volume solely results in an increase in the liquid level, with minimal impact on other indicators.

### 3.2. Results of Suspensions

#### 3.2.1. Comparison with the OCS-AMS for AC Suspension with Relatively Stable Absorbance

**Figure 8:**
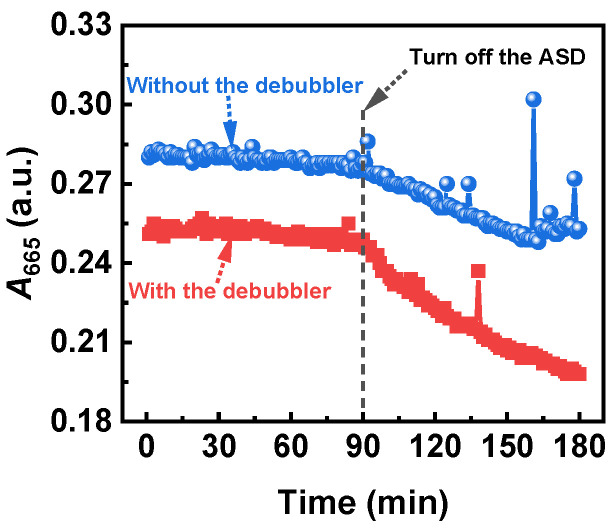
Time-dependent *A*_665_ of the AC suspension with relatively stable absorbance, both before and after the ASD is turned off without and with the debubbler.

To demonstrate the effectiveness of the proposed ASD in preventing PDP, we used the AC suspension with relatively stable absorbance after prolonged stirring (stirring continuously for over 24 h at room temperature) as a comparative case study. Figure 8 presents a comparison of the absorbance variation at a wavelength of 665 nm (*A*_665_) of the AC suspension before and after the ASD is turned off, with and without the debubbler, respectively. As depicted in the figure, once the ASD is turned off, the absorbance quickly begins to decrease, regardless of the presence or absence of the debubbler. Specifically, as demonstrated in the figure, in the absence of the debubbler, there is a minimal decrease in absorbance prior to turning off the ASD, and no observable SNB is present. However, after the ASD is turned off, a significant decrease in absorbance is accompanied by visible SNB. The rates of the absorbance decrease are 5.2 × 10^−5^ and 4.0 × 10^−4^ (a.u./min) when the ASD is active and inactive, respectively. This suggests the effectiveness of the ASD in eliminating SNB and PDP, highlighting the significant practical importance of the ASD for online circulatory monitoring for SPBRs.

Moreover, as illustrated in the figure, the *A*_665_ trend with the debubbler is generally in line with the scenario without the debubbler when the ASD is active; however, there is a more rapid decrease in absorbance when the ASD is deactivated. Specifically, the rate of absorbance reduction when the ASD is active is 5.2 × 10^−5^ (a.u./min), whereas the initial rate of absorbance reduction when the ASD is inactive is 1.4 × 10^−3^ (a.u./min). The accelerated decline in absorbance after ASD deactivation may be attributed to the smoother downstream flow facilitated by the debubbler, promoting PDP. When the ASD is activated, its high-speed slug flow can minimize the influence of the debubbler. This indicates that the ASD not only effectively mitigates PDP but also synergizes effectively with the debubbler. Consequently, the integration of ASD and the debubbler holds significant practical value in enabling online automated monitoring for SPBRs.

#### 3.2.2. Comparison with the OCS-AMS for TiO_2_ Suspension

**Figure 9:**
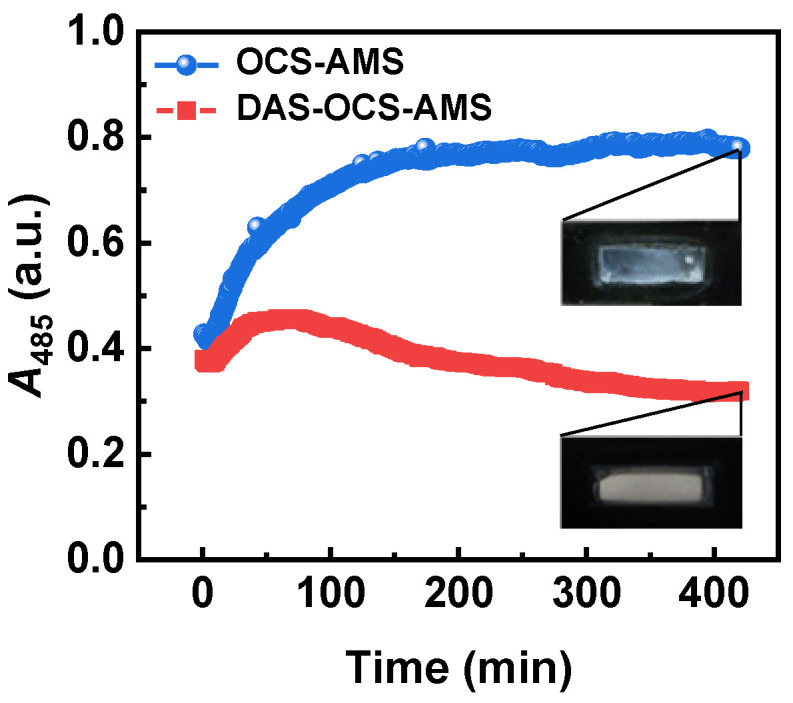
The time-dependent *A*_485_ of TiO_2_ suspension obtained by the OCS-AMS and the DAS-OCS-AMS.

For a more intuitive assessment of the effectiveness of the DAS-OCS-AMS in preventing PDW, Figure 9 compares the time-dependent absorbance at 485 nm (*A*_485_) between the OCS-AMS and the DAS-OCS-AMS (with a 1 min *TR* and 10 s *AD*). As depicted in the figure, the DAS-OCS-AMS demonstrates significantly superior performance in preventing PDW compared to the OCS-AMS. Specifically, the results obtained from the OCS-AMS show a continual increase in absorbance over time due to the direct accumulation of PDW, resulting in inaccurate monitoring outcomes. Additionally, the inset at the end of the automated monitoring period reveals visible white TiO_2_ deposition on the flow cell window, indicating that the absorbance of suspended TiO_2_ solid particles cannot be accurately measured by the OCS-AMS. In contrast, the data from the proposed DAS-OCS-AMS indicate a brief rise in absorbance during monitoring, followed by a decline. At the conclusion of the automated monitoring period, minimal PDW is observed, as shown in the inset. This result is primarily attributed to the operation of the ASD, which generates high-velocity slug flow to swiftly remove the PDW, thereby significantly reducing its formation. This underscores that the integration of the ASD in the proposed DAS-OCS-AMS can effectively alleviate the impact of PDW.

#### 3.2.3. Three-Wavelength Monitoring Results of Three Suspensions with Different TR

**Figure 10:**
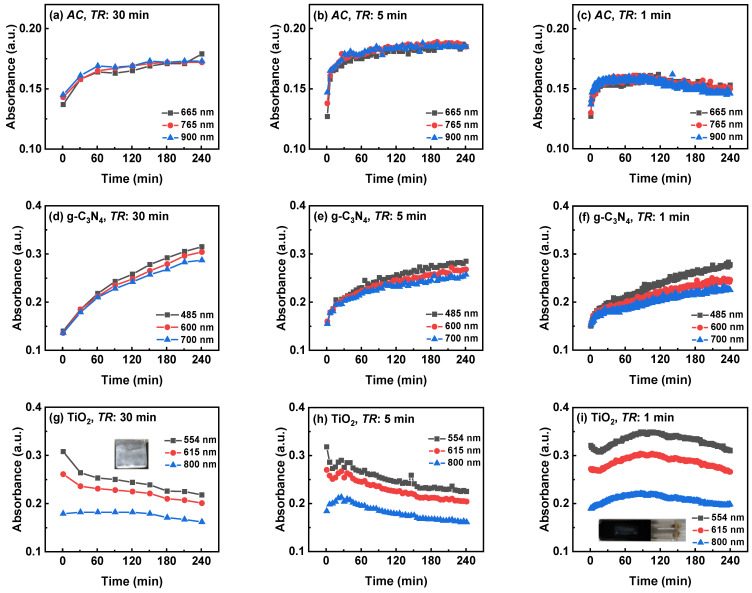
The time-dependent absorbance of three suspensions of (**a**–**c**) AC, (**d**–**f**) g-C3N4, and (**g**–**i**) TiO_2_ at three *TR* (30, 5, and 1 min) and with 10 s *AD*.

To further investigate the preventive effect of PDP and PDW by DAS-OCS-AMS, and to demonstrate the time-dependent absorbance characteristics of different suspensions, we analyzed the influence of *TR* on the changes in absorbance for three types of suspension. Figure 10 illustrates the time-dependent absorbance at three different wavelengths for three suspensions under stirring, with three different *TR* values (30, 5, and 1 min) and 10 s *AD*. As shown in the figure, the absorbance trends over time may differ among different suspensions. For instance, taking the results of 30 min *TR* for each suspension, both AC and g-C_3_N_4_ show continuous enhancement, while that of TiO_2_ becomes weaker. This is likely due to the disagglomeration of AC and g-C_3_N_4_ suspensions under stirring, while the TiO_2_ suspension experiences aggregation near the isoelectric point and deposition on the inner wall of the reactor. This indicates that different suspensions may exhibit different trends in optical properties, and, therefore, the proposed DAS-OCS-AMS can monitor the optical properties of the suspensions.

Furthermore, it is evident that the results with a 30 min *TR* can reflect the intrinsic absorbance changes in the suspensions. For example, the results of the TiO_2_ suspension show no significant PDW phenomenon, only reactor deposition. This is because when *TR* is 30 min, the total circulation times of the suspension in the monitoring system are low, resulting in a small cumulative effect of PDW. Therefore, the results with a 30 min *TR* provide a valuable baseline for evaluating the results of the other *TR* values.

Moreover, comparing the results obtained with the other two *TR* to those with a 30 min *TR* reveals that, as the values of *TR* decrease, the impact of PDP or PDW on different suspensions is not the same. Specifically, the influence on TiO_2_ is the greatest, followed by AC, and the g-C_3_N_4_ suspension shows the least impact. This may be attributed to the different properties of the suspensions, including their agglomeration and disaggregation, as well as their interactions with the reactor, pipelines, and flow cell windows. It is important to note the limitation of this approach in relation to the morphological and textural properties of the photocatalysts. Therefore, choosing a lower *TR* based on the reaction time can minimize the impact of PDP and PDW on the results, providing valuable insights for determining system parameters to ensure greater accuracy and precision.

#### 3.2.4. Three-Wavelength Monitoring Results of TiO_2_ Suspension with Different AD

**Figure 11:**
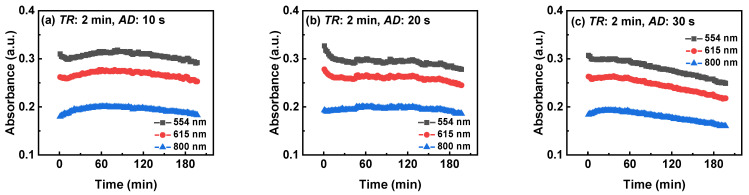
The time-dependent absorbance of the TiO_2_ suspension at the three wavelengths (554, 615, and 800 nm) under three *AD* conditions ((**a**) 10, (**b**) 20, and (**c**) 30 s) with a 2 min *TR*.

To further analyze whether extending the *AD* can enhance the preventive effect of PDW, Figure 11 illustrates the temporal evolution of absorbance at three wavelengths for the TiO_2_ suspension under three *AD* conditions (10, 20, and 30 s) with a 2 min *TR*. As shown in the figure, extending the *AD* can enhance the preventive effect of PDW. Specifically, a comparison between Figure 10i and Figure 11a clearly indicates that a slight reduction in *TR* alone is insufficient to effectively diminish the impact of PDW. In contrast, at an *AD* of 30 s, the trend closely aligns with the results at a 30 min *TR* in Figure 10g. This is attributed to the extension of the *AD*, which lengthens the duration of slug flow action, highlighting the significant role of prolonging the action time of slug flow in preventing PDW. Therefore, practical applications should involve adjustments to *TR* and *AD* in accordance with the reaction characteristics and reaction time to mitigate the influence of PDW.

### 3.3. Comparison with the OCS-AMS for the Photocatalytic Degradation of AOII by TiO_2_

#### 3.3.1. Results under Visible Illumination

**Figure 12:**
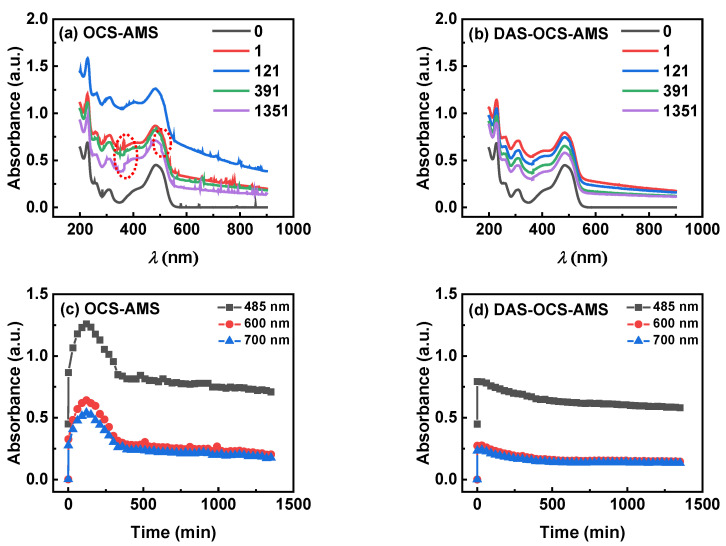
The representative UV-visible absorption spectra of the photocatalytic degradation of AOII by TiO_2_, obtained using (**a**) OCS-AMS and (**b**) DAS-OCS-AMS under visible LED illumination. The corresponding temporal variation in absorbance at three wavelengths, as measured by (**c**) OCS-AMS and (**d**) DAS-OCS-AMS.

To demonstrate the advantages of DAS-OCS-AMS in monitoring suspended photocatalytic reactions, particularly its ability to accurately and simultaneously acquire absorbance of both the mixture and solid particles, Figure 12 compares the stability of the UV-visible absorption spectra over time during the monitoring of the photocatalytic degradation of AOII by TiO_2_ under visible illumination using the OCS-AMS and DAS-OCS-AMS.

Upon closer examination of the UV-visible absorption spectra obtained by OCS-AMS (as shown in Figure 12a), significant SNB and large jumps in absorbance can be observed, particularly occurring when the wavelength shifts from 506 nm to 505 nm and from 367 nm to 366 nm. These jumps are likely due to the effects of PDW on the monitoring signal. Additionally, as shown in Figure 12c, the plots of absorbance of the three wavelengths exhibit a rollercoaster-like shape, with the maximum value occurring at 121 min. These occurrences are associated with strong adsorption and competition between TiO_2_ solid particles and the reactor, pipeline, and flow cell windows. This suggests that utilizing only the simple OCS-AMS may not be able to obtain smooth absorption spectra and accurate results for the mixture and suspended solid particles.

Conversely, the UV-visible absorption spectra obtained by the proposed DAS-OCS-AMS are exceptionally smooth, exhibiting minimal SNB and jumps, as shown in Figure 12b. Additionally, the intensity of the absorption spectra gradually decreases over time. Specifically, as shown in Figure 12d, the maximum absorption spectra intensity occurs at 1 min. This is because the debubbler and the ASD of the DAS-OCS-AMS each play their roles in eliminating SNB and preventing PDP and PDW, thereby reflecting the effect of particle deposition on the inner wall of the reactor.

Moreover, the shift in absorbance towards larger values in the near-infrared region when the OCS-AMS and DAS-OCS-AMS are not equal to “0” can be attributed to the increased scattering and absorption of light by the particles in the suspension. This effect is more pronounced in the OCS-AMS system compared to the DAS-OCS-AMS due to the additional noise and interference of PDW present in the former system. The DAS-OCS-AMS, with its ASD to mitigate the issue of PDW, provides more accurate and reliable absorbance measurements in the presence of suspended solid particles. This indicates that the DAS-OCS-AMS can accurately obtain the absorbance of both the mixture and suspended solid particles.

#### 3.3.2. Results under UV Illumination

**Figure 13:**
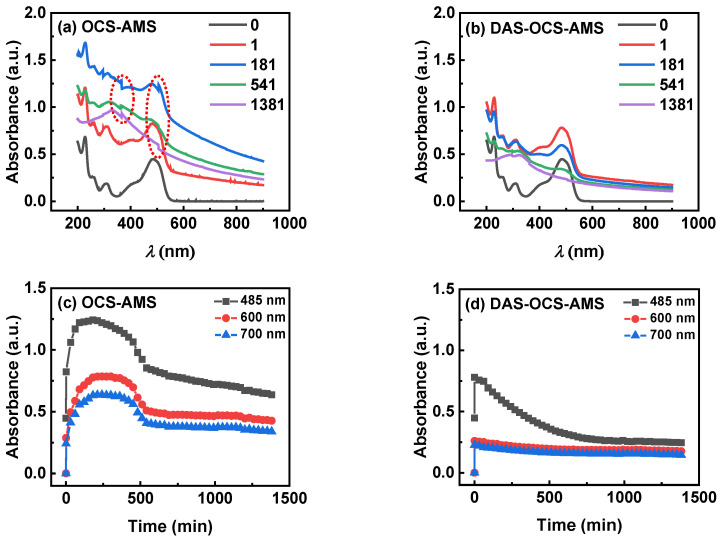
The representative UV-visible absorption spectra of the photocatalytic degradation of AOII by TiO_2_, obtained using (**a**) OCS-AMS and (**b**) DAS-OCS-AMS under UV LED illumination. The corresponding temporal variation in absorbance at three wavelengths, as measured by (**c**) OCS-AMS and (**d**) DAS-OCS-AMS.

To further compare the performance of OCS-AMS and DAS-OCS-AMS in the automated monitoring of the photocatalytic degradation of AOII by TiO_2_ under UV illumination, the corresponding automated monitoring results are presented in Figure 13. As illustrated, the results from the OCS-AMS are also significantly affected by PDW, and the results from the DAS-OCS-AMS are insignificant. Specifically, as shown in Figure 13a,c, they not only exhibit the same overall intensity changes in the absorption spectra but also display notable signal jumps. This again indicates that using OCS-AMS is unable to provide accurate information on the absorbance of the mixture and the TiO_2_ solid particles. In contrast, as shown in Figure 13b,d, the results from the DAS-OCS-AMS do not exhibit the rollercoaster or jumpy patterns. These characteristics make the DAS-OCS-AMS an efficient and reliable online monitoring system, suitable for studying the kinetics of SPBRs from both the mixture and solid particle dimensions simultaneously.

Both Figure 13b,d depict a rapid decrease in absorbance at the maximum absorption wavelength (λ_max_) 485 nm of AOII, while the intensity in the non-absorption range of AOII (wavelengths > 600 nm) gradually decreases. This observation signifies the system’s capacity to simultaneously monitor changes in the absorbance of the mixture and solid particles. Furthermore, prior research [21] has demonstrated the additivity of the contributions of the absorbance of the substrate and solid particles. Consequently, it can be inferred that estimating the absorbance of the solid particles at the λ_max_ of the substrate based on the absorbance of the solid particles in the non-absorption range of the substrate would allow for the determination of the absorbance of the substrate using additivity. This implies that, through data processing, kinetic data for the substrate can be derived from the data obtained from the proposed system. Consequently, if the aforementioned data processing method is validated, the proposed hardware system and data processing method will have significant practical implications in photocatalyst fabrication, reactor design, and comprehension of the mechanism of SPBRs [7].

## 4. Conclusions

In this study, we proposed an innovative online circulatory automated monitoring system, known as the DAS-OCS-AMS, for studying the kinetics of SPBRs. We demonstrate how incorporating both the debubbler and ASD into the OCS-AMS can minimize the influence of SNB, PDW, and PDP, and how optimizing the parameters can enhance the accuracy and robustness of the DAS-OCS-AMS. Initially, the OCS-AMS was used to ensure operational simplicity and to avoid interference from excitation light. Then, we constructed a debubbler to reduce the impact of SNB. Third, we developed an ASD to mitigate the influence of PDW and PDP. This is based on the rationale that the high-speed slug flow possesses a powerful cleaning effect for PDW, PDP, and bubbles and that the reduction in total circulation time can reduce the influence of PDW and PDP. Additionally, we delved into analyzing the impact of the parameters on the performance of the DAS-OCS-AMS. Finally, a photocatalytic reaction was conducted, and the results indicate that the DAS-OCS-AMS can accurately monitor the absorbance of the mixture and solid particles.

Notably, although this study only used a spectrophotometer as a detector to validate the effectiveness of the DAS-OCS-AMS, the proposed system represents a highly flexible universal framework for online circulatory automated monitoring with additional optical detectors, such as Raman and infrared spectrometers. By replacing, using in series, or paralleling with these optical detectors, researchers will be able to obtain kinetic data of SPBRs from multiple perspectives, contributing to a deeper understanding of reaction mechanisms [39].

However, the validation of the proposed DAS-OCS-AMS in this study is confined to the stability and accuracy of both the absorbance of the mixture and the solid particles, while the data processing method requires future research attention. By incorporating the data processing method, we can obtain the absorbance data of the substrate. Future work will focus on developing and validating data processing methods that take into account the characteristics of absorbance spectra of mixture suspensions and on employing deconvolution or three-wavelength estimation models to obtain kinetic data for the substrate.

## Data Availability

Data are available on request.

## Supplementary Materials

The following supporting information can be downloaded at: https://www.mdpi.com/article/10.3390/nano14060508/s1; Table S1: Experimental conditions and parameters for the water case studies; Table S2: Experimental conditions and parameters for the case studies of pure solid aqueous suspensions; Table S3: Experimental conditions and parameters for the case studies of the photocatalytic degradation of AOII by TiO_2_.
