# Supplementary material for: Automated Monitoring System for Suspended Photocatalytic Batch Reactions Based on Online Circulatory Spectrophotometry
Source: Nanomaterials (Basel). 2024 Mar 12;14(6):508. doi: 10.3390/nano14060508 (PMC10975201; doi:10.3390/nano14060508)
Supplement: Supplementary file 1 [file nanomaterials-14-00508-s001.zip › nanomaterials-2906089-supplementary.pdf]

Supplementary Materials

Automated Monitoring System for Suspended Photocatalytic Batch Reactions Based on Online Circulatory Spectrophotometry

Da-Peng Lei [1,2] and Jian-Hua Huang [1,*]

[1] Department of Chemistry, Zhejiang Sci-Tech University, Hangzhou 310018, China; photocatalyst@163.com
[2] Wenzhou Quality and Technology Testing Research Institute, Wenzhou 325007, China
* Correspondence: jhhuang@zstu.edu.cn

Table S1. Experimental conditions and parameters for the water case studies

Units	Experimental conditions and parameters	**3.1.1. Comparison of Signal Stability under Two Severe Experimental Conditions**				**3.1.2. Analysis of the Mechanism of SNB Generation in the OCS-AMS**				**3.1.3. Influence of Parameters on the Operational Stability of the Debubbler**			
		**5(a) left**	**5(a)right**	**5(b) left**	**5(b)right**	**6(a)**	**6(b)**	**6(c)**	**6(d)**	**7(a-c)**	**7(d-f)**	**7(g-i)**	**7(j-l)**
Reactor unit	Reactor	Quartz photocatalytic reactor (250 mL)				Quartz photocatalytic reactor (250 mL)				Quartz photocatalytic reactor (250 mL)			
	Volume of water (mL)	200				200				200			
	Stir bar (mm*mm)	A50 (17*50)		B25 (8*25)		——	A50 (17*50)	A50 (17*50)	B25 (8*25)	B25 (8*25)	B25 (8*25)	B25 (8*25)	A50 (17*50)
	Stirring speed (rpm)	1400		750		——	500, 750, 1000, 1100, 1200, 1400	1400	750	750	750	750	500, 750, 1000, 1200, 1400
	Temperature (K)	295		364		295	295	295	295, 325, 338, 347, 355, 366	296	297, 314, 328, 343, 358	297	295
Segmented and aerated flow unit	Off time of intermittent timer (s)	——				——				1			

Units	Experimental conditions and parameters	**3.1.1. Comparison of Signal Stability under Two Severe Experimental Conditions**				**3.1.2. Analysis of the Mechanism of SNB Generation in the OCS-AMS**				**3.1.3. Influence of Parameters on the Operational Stability of the Debubbler**			
		**5(a) left**	**5(a)right**	**5(b) left**	**5(b)right**	**6(a)**	**6(b)**	**6(c)**	**6(d)**	**7(a-c)**	**7(d-f)**	**7(g-i)**	**7(j-l)**
	On time of intermittent timer (s)	— —				— —				59			
	Delay time of aeration delay relay (s)	— —				— —				40			
	Delay time of drainage delay relay (s)	— —				— —				10			
Debubble unit	With or without debubble unit	without	with	without	with	without				with			
	Syringe volume (mL)	— —	36	— —	65	— —				36	36	8, 19, 28, 36, 46, 54	36
Detection unit	Mode	Time scan				Time scan				Time scan			
	TR (s)	1				1				1			
	Interval (s)	1				1				1			
	Duration (s)	600				600				600			
	Wavelength (nm)	600				600				600			
Online circulation unit	Circulation velocity (mL/min)	71				18, 34, 54, 66, 71	18	18, 34, 54, 66, 71	45	18, 27, 34, 45, 54, 61, 66, 71	61	61	61

Table S2. Experimental conditions and parameters for the case studies of pure solid aqueous suspensions

Units	Experimental conditions and parameters	3.2.1. Comparison with the OCS-AMS for AC Suspension with Relatively Stable Absorbance		3.2.2. Comparison with the OCS-AMS for TiO_2 Suspension		3.2.3. Three-wavelength Monitoring Results of Three Suspensions with Different *TR*									3.2.4. Three-wavelength Monitoring Results of TiO_2 Suspension with Different *AD*	
		**8(a)**	**8(b)**	**9(a)**	**9(b)**	**10(a)**	**10(b)**	**10(a)**	**10(d)**	**10(e)**	**10(f)**	**10(g)**	**10(h)**	**10(i)**	**11(a)**	**11(b)**
Reactor unit	Reactor	Quartz photocatalytic reactor (250 mL)														
	volume of water (mL)	200		200		200			200			200			200	
	Solid particles	AC		TiO_2		AC			$g\text{-}C_3N_4$			TiO_2			TiO_2	
	Mass concentration (mg/L)	150		50		150			150			150			50	
	Stir bar (mm*mm)	B25 (8*25)		B25 (8*25)		B25 (8*25)			B25 (8*25)			B25 (8*25)			B25 (8*25)	
	Stirring speed (rpm)	750		750		750			750			750			750	
	Temperature (K)	Room temperature		Room temperature		Room temperature			Room temperature			Room temperature			Room temperature	
Segmented and aerated flow unit	Off time of intermittent timer (s)	1		1		1741	241	1	1741	241	1	1741	241	1	61	
	On time of intermittent timer (s)	59		59		59			59			59			59	
	Delay time of aeration delay relay (s)	40		40		40			40			40			40	
	Delay time of drainage delay relay (s)	10		10		10			10			10			10	20
Debubble unit	With or without debubble unit	Without	With	Without	With	With			With			With			With	

Units	Experimental conditions and parameters	3.2.1. Comparison with the OCS-AMS for AC Suspension with Relatively Stable Absorbance		3.2.2. Comparison with the OCS-AMS for TiO_2 Suspension		3.2.3. Three-wavelength Monitoring Results of Three Suspensions with Different *TR*									3.2.4. Three-wavelength Monitoring Results of TiO_2 Suspension with Different *AD*	
		**8(a)**	**8(b)**	**9(a)**	**9(b)**	**10(a)**	**10(b)**	**10(a)**	**10(d)**	**10(e)**	**10(f)**	**10(g)**	**10(h)**	**10(i)**	**11(a)**	**11(b)**
	Syringe volume (mL)	——	10	——	10	10			10			10			36	
Detection unit	Mode	Photometric measurement		Photometric measurement		Photometric measurement			Photometric measurement			Photometric measurement			Photometric measurement	
	TR (min)	1		1		30	5	1	30	5	1	30	5	1	2	
	Interval (s)	44.64		45.20		1784.64	284.64	44.64	1785.20	285.20	45.20	1784.36	284.36	44.36	104.36	
	Duration (min)	180		400		240			240			240			185	
	Wavelengths (nm)	665, 765, 900		485, 600, 700		665, 765, 900			485, 600, 700			554, 615, 800			554, 615, 800	
Online circulation unit	Circulation velocity (mL/min)	45		45		45			45			45			61	

Table S3. Experimental conditions and parameters for the case studies of the photocatalytic degradation of AOII by TiO_2

Units	Experimental conditions and parameters	3.3.1. Results under Visible Illumination		3.3.2. Results under UV Illumination	
		12(a) and 12(c)	12(b) and 12(d)	13(a) and 13(c)	13(b) and 13(d)
Reactor unit	Reactor	Four-necked glass flask (250 mL)		Quartz photocatalytic reactor (250 mL)	
	Volume of water (mL)	199		199	
	Solid particles	TiO_2		TiO_2	
	Mass concentration of solid particles (mg/L)	50		50	
	Substrate	AOII		AOII	
	Volume of substrate stock solution added (mL)	1.00		1.00	
	Mass concentration of substrate (mg/L)	10		10	
	Stir bar (mm*mm)	B25 (8*25)		B25 (8*25)	
	Stirring speed (rpm)	750		750	
	Temperature (K)	303		Room temperature	
	Light source	4 visible LED lamps		1 UV LED lamp	
	Dark adsorption time (min)	60		60	
Segmented and aerated flow unit	Off time of intermittent timer (s)	——	1470	——	1470
	On time of intermittent timer (s)	——	330	——	330
	Delay time of aeration delay relay (s)	——	250	——	250
	Delay time of drainage delay relay (s)	——	60	——	60
Debubble unit	With or without debubble unit	Without	With	Without	With
	Syringe volume (mL)	——	36	——	36
Detection unit	Mode	Spectral scan		Spectral scan	Spectral scan
	TR (min)	30		30	30
	Interval (s)	1600		1600	
	Duration (min)	1351		1381	
	Wavelengths (nm)	200 ~ 900		200 ~ 900	
Online circulation unit	Circulation velocity (mL/min)	61		61	
